# Recent-onset and persistent tinnitus: Uncovering the differences in brain activities using resting-state functional magnetic resonance imaging technologies

**DOI:** 10.3389/fnins.2022.976095

**Published:** 2022-10-19

**Authors:** Haoliang Du, Xu Feng, Xiaoyun Qian, Jian Zhang, Bin Liu, Ao Li, Zhichun Huang, Xia Gao

**Affiliations:** ^1^Jiangsu Provincial Key Laboratory Medical Discipline, Department of Otolaryngology-Head and Neck Surgery, Affiliated Drum Tower Hospital of Nanjing University Medical School, Nanjing, China; ^2^Department of Research Institution of Otolaryngology, Affiliated Drum Tower Hospital of Nanjing University Medical School, Nanjing, China; ^3^Department of Otolaryngology-Head and Neck Surgery, Zhongda Hospital, Medical School, Southeast University, Nanjing, China; ^4^Department of Radiology, Zhongda Hospital, Southeast University, Nanjing, China

**Keywords:** recent-onset tinnitus, persistent tinnitus, resting-state functional magnetic resonance imaging, amplitude of low-frequency fluctuation, regional homogeneity, voxel-wise functional connectivity

## Abstract

**Objective:**

This study aimed to investigate the differences in intra-regional brain activity and inter-regional functional connectivity between patients with recent-onset tinnitus (ROT) and persistent tinnitus (PT) using resting-state functional magnetic resonance imaging (rs-fMRI), including the amplitude of low-frequency fluctuations (ALFF), regional homogeneity (ReHo), and voxel-wise functional connectivity (FC).

**Method:**

We acquired rs-fMRI scans from 82 patients (25 without recent-onset tinnitus, 28 with persistent tinnitus, and 29 healthy controls). Age, sex, and years of education were matched across the three groups. We performed ALFF, ReHo, and voxel-wise FC analyses for all patients.

**Results:**

Compared with the control group, participants with ROT and PT manifested significantly reduced ALFF and ReHo activity within the left and right dorsolateral superior frontal gyrus (SFG) and gyrus rectus (GR). Additional voxel-wise FC revealed decreased connectivity between the dorsolateral SFG (left and right) and the right superior parietal gyrus (SPG), right middle frontal gyrus (MFG), and left medial superior frontal gyrus (mSFG) within these two groups. Significant differences were observed between the ROT and PT groups, with the ROT group demonstrating reduced FC.

**Conclusion:**

Our data suggest that patients with PT have more difficulty monitoring external stimuli and reorienting attention than patients with ROT. In addition, patients who perceive higher levels of disruption from tinnitus are more likely to develop persistent and debilitating tinnitus once the tinnitus lasts longer than six months. Therefore, we strongly recommend that clinicians implement effective tinnitus management strategies in patients with ROT as soon as possible.

## Introduction

Subjective tinnitus is a conscious auditory perception without a corresponding external source and is one of the most common yet distressing otologic pathologies, affecting approximately 8–20% of the adult population (Roberts et al., 2010). Studies have reported that subjective tinnitus is commonly associated with hearing loss, cerumen impaction, middle and inner ear-related pathologies, noise exposure, exposure to ototoxic medications and chemicals, aging, insomnia, anxiety, depression, head and neck injuries, and temporomandibular joint (TMJ) dysfunction (Baguley et al., 2013; Tunkel et al., 2014; Makar, 2021). In addition, tinnitus can be persistent, bothersome, and costly for patients and society in general. Cases of patients with extraordinarily persistent and debilitating tinnitus accompanied by severe anxiety or depression attempting suicide have been reported (Szibor et al., 2019).

It is generally believed that lesions in the peripheral hearing system and neuronal changes in the central nervous system contribute to tinnitus. Kapolowicz and Thompson (2020) reported that tinnitus might be closely related to an imbalance between auditory neuronal excitation and the inhibition network, leading to plasticity changes in the central auditory system. Knipper et al. (2021) proposed that hearing loss may contribute to a top-down mechanism that leads to tinnitus perception (Knipper et al., 2021). Khan et al. (2021) suggested that tinnitus might be a compensatory response to peripheral hearing system damage (Khan et al., 2021). Cai et al. (2020) reported abnormal functional connectivity (FC) in the auditory and non-auditory cortices in patients with hearing loss and tinnitus (Cai et al., 2020). Zhou et al. (2019) suggested that patients with hearing loss and tinnitus demonstrate abnormal intra-regional neural activity and disrupted connectivity in the hub regions of some non-auditory networks, including the default mode network (DMN), optical network, dorsal and ventral attention network (DAN and VAN), and central executive network (CEN) (Zhou et al., 2019). Minami et al. (2018) reported that tinnitus patients with hearing loss showed a statistically significant reduction in auditory-related FC compared with the control group. Finally, our previous project, using amplitude of low-frequency fluctuations (ALFF), regional homogeneity (ReHo), and voxel-wise functional connectivity (FC) technologies, revealed that disruptions in the brain regions responsible for attention and stimuli monitoring and orientations could lead to tinnitus. Tinnitus has different forms, degrees of severity, and onset duration, which can only be described by patients’ testimony and corresponding symptoms. When categorizing tinnitus based on its onset duration (recent-onset or persistent), numerous studies have concentrated on developing pathophysiological models for chronic tinnitus (tinnitus that has an onset duration of at least six months). However, few studies have investigated the neuronal changes that occur from recent-onset to persistent tinnitus (PT) (Stolzberg et al., 2013; Cai et al., 2020; Lan et al., 2020). To the best of our knowledge, no studies have investigated the aforementioned issue using resting-state functional magnetic resonance imaging (rs-fMRI) technologies. Furthermore, investigating this issue is critical for identifying the contributing neural mechanisms and possible interventions to stop this transition. Therefore, our project aims were to uncover the differences in brain activity between recent-onset tinnitus (ROT) patients and PT patients using resting-state functional magnetic resonance imaging (rs-fMRI) technologies and to apply our findings to existing tinnitus management strategies.

## Materials and methods

### Participants’ demographic and clinical information

This study was approved by the Research Ethics Committee of the Affiliated Zhongda Hospital, Southeast University. All participants provided written informed consent before participating in the study. We recruited 82 participants (all right-handed, with at least eight years of education), including 25 tinnitus participants with recent-onset tinnitus (ROT), 28 tinnitus participants with persistent tinnitus (PT), and 29 healthy participants as the control group, through our outpatient clinics between September 2011 and September 2013. The patients were group matched in terms of age, sex, and education level. Twenty-five participants perceived bilateral tinnitus and the remaining, 28 participants, perceived unilateral tinnitus. We defined the time course of tinnitus (recent-onset or persistent) according to the Tinnitus Clinical Practice Guidelines of the American Academy of Otolaryngology-Head and Neck Surgery. According to the guideline, if the overall duration of onset equals or is less than six months, tinnitus will be determined to be recent-onset. If the overall duration of onset is more than six months, tinnitus will be defined as persistent (Tunkel et al., 2014).

We performed pure-tone audiometric testing (PTA at 250; 500; 1,000; 2,000; 4,000; 6,000, and 8,000 Hz) for all recruited participants. Participants with a 7-frequency PTA < 25 dB HL were considered to have clinically normal hearing. In addition, we performed comprehensive tympanometry, diagnostic distortion-product otoacoustic emission (DPOAE), and diagnostic auditory brainstem response (ABR) to rule out middle ear pathologies and auditory neuropathy (ANSD). Furthermore, we collected crucial information about the duration of tinnitus and presence of insomnia in all participants.

To assess the distress associated with tinnitus, we distributed the Iowa version of the tinnitus handicap questionnaire (THQ) to both the ROT and PT groups. We also distributed the Self-Rating Depression Scale (SDS) and Self-Rating Anxiety Scale (SAS) questionnaires to all participants for anxiety and depression screening (Zung, 1971). No significant group differences were found in the patients’ gender, age, and educational background (*p* > 0.05). However, we found a statistically significant difference in the THQ total score, SAS, and SDS scores between the groups (*p* < 0.05). The demographic and clinical characteristics of the participants in each group are summarized in Table 1.

**TABLE 1:** Subject characteristics of the ROT, PT, and control group.

	ROT group	PT group	Control group	*P*-value
Age (year)	45.32 ± 2.93	42.68 ± 2.33	37.38 ± 1.84	>0.05
Gender (male: female)	14:11	14:14	20:9	>0.05
Education duration (year)	9.83 ± 2.11	9.22 ± 1.96	10.12 ± 2.43	>0.05
THQ total score	40.67 ± 3.89	44.97 ± 4.27	–	<0.05
SAS score	35.12 ± 1.07	37.57 ± 1.51	–	<0.05
SDS score	37.96 ± 1.85	39.06 ± 2.12	–	<0.05

Data are represented as Mean ± SD. ROT, recent-onset tinnitus; PT, persistent tinnitus.

### Subject exclusion criteria

The exclusion criteria for this study included Meniere’s disease, objective tinnitus, pulsatile tinnitus, history of alcohol consumption, severe smoking, head and neck injuries, epilepsy, stroke, Alzheimer’s disease, Parkinson’s disease, cancer, MRI contraindications, primary psychiatric conditions including generalized anxiety disorder (GAD), depression, schizophrenia, and severe visual impairment. None of our participants failed depression or anxiety screening.

### Functional magnetic resonance imaging scanning and data acquisition

Imaging data using a 3.0 T MRI scanner (Siemens MAGENETOM Trio, Erlangen, Germany) with a standard head coil. All participants were provided with foam paddings and earmuffs to minimize head motion and noise exposure during the scanning process. The participants were instructed to remain calm during the scan with their eyes closed, without falling asleep or thinking of anything particular. Functional images were obtained axially using a gradient echo-planar sequence sensitive to BOLD contrast, as follows: repetition time (TR), 2,000 ms; echo time (TE), 25 ms; slices, 36; thickness, 4 mm; gap, 0 mm; field of view (FOV), 240 mm × 240 mm; acquisition matrix, 64 × 64; and flip angle (FA), 90°.

### Amplitude of low-frequency fluctuations: Pre-processing and analysis

Resting-state ALFF reflects spontaneous neural activity and yields physiologically meaningful results. Pre-processing of the ALFF images was performed using the Data Processing Assistant for Resting-State fMRI (DPARSF 5.2), Statistical Parametric Mapping (SPM12), and MATLAB 2021b. The first ten volumes were removed from each time series to account for the participants’ adaptation to the scanning environment. Slice timing and realignment for head motion correction were performed on the remaining 175 images. Subsequently, we performed the following procedures: spatially normalized into the stereotactic space of the Montreal Neurological Institute (MNI) (resampling voxel size = 3 Ã–3 Ã–3 mm^3^) and smoothed using a Gaussian kernel of 6 mm full width at half-maximum (FWHM), de-trending, and filtering (0.01–0.08 Hz). The participants with a head motion of more than 2.0 mm displacement or a 2.0-degree rotation in the x, y, or z directions were excluded from this study.

We then analyzed the ALFF data by transforming the time domain to the frequency domain using a fast Fourier transform. Next, we computed the square root of the power spectrum and averaged squared across 0.01–0.08 Hz at each voxel. The calculated average square root was used as the ALFF. Finally, the ALFF of each voxel was divided by the global mean ALFF value for standardization.

### Regional homogeneity: Pre-processing and analysis

Regional homogeneity calculates the synchronization of low-frequency fluctuations between a given voxel and neighboring voxels, reflecting the neural function synchronization in the local brain region. Pre-processing of ReHo images was performed using DPARSF 5.2, SPM12, and MATLAB 2021b. The first ten volumes were removed from each time series to account for the participants’ adaptation to the scanning environment. Slice timing and realignment for head motion correction were performed on the remaining 175 images. The following procedures were performed: spatial normalization into the stereotactic space of the MNI (resampling voxel size = 3 Ã–3 Ã–3 mm^3^), de-trending, and filtering (0.01–0.08 Hz).

After the pre-processing stage, we performed the image calculation using the Kendall coefficient of concordance of the time series of a given voxel with its 27 nearest neighbors. Next, ReHo analyses were performed using DPARSF 5.2 software. The ReHo value of each voxel was standardized by partitioning the primal value using the global mean ReHo value. Finally, the data were smoothed with a Gaussian kernel of 6 mm FWHM for further statistical analysis.

### Voxel-wise functional connectivity: Pre-processing and analysis

We performed voxel-wise FC analysis using DPARSF 5.2, SPM12, and MATLAB 2021b. The first ten volumes were removed from each time series to account for the participants’ time to adapt to the scanning environment. Slice timing and realignment for head motion correction were then performed for the remaining 170 images. Subsequently, the procedures were carried out as follows: spatially normalized into the stereotactic space of the MNI (resampling voxel size = 3 Ã–3 Ã–3 mm^3^), and smoothed using a Gaussian kernel of 6 mm FWHM, de-trending, and filtering (0.01–0.08 Hz). Participants with a head motion with more than 2.0 mm displacement or a 2.0-degree rotation in the x, y, or z directions were excluded.

We extracted the ALFF and ReHo differences in brain regions between ROT participants and PT participants for voxel-wise FC analysis and defined them as seeds. We then used the average time series of seeds as a reference and calculated the Pearson correlation coefficient between the average signal change of each seed and the time sequences of the other voxels in the brain. Finally, we converted the correlation coefficient to a z-value by using Fisher’s z-transformation.

### Statistical analysis and graphic illustration

One-way analysis of variance (ANOVA) was first conducted to test the mean differences in ALFF, ReHo, and FC between the CG, the ROT group, and the group with PT (MATLAB 2021b). Statistically significant differences between groups were determined at *p* < 0.05. The participants’ age and sex were included as nuisance covariates. Next, we applied family wise error (FWE) correction for multiple comparisons using voxel-level inference at *p* < 0.001 and cluster-level inference at *p* < 0.05. Two-sample t-tests were then conducted to investigate the ALFF, ReHo, and FC differences between participants with ROT and the control group, participants with PT and the control group, and participants with ROT and participants with PT. Again, a statistically significant difference between groups was determined at *p* < 0.05. Finally, we used MRIcroGL software to draw 2-dimensional brain images to display the brain areas with statistically significant differences.

## Results

### Amplitude of low-frequency fluctuations results

**FIGURE 1:**
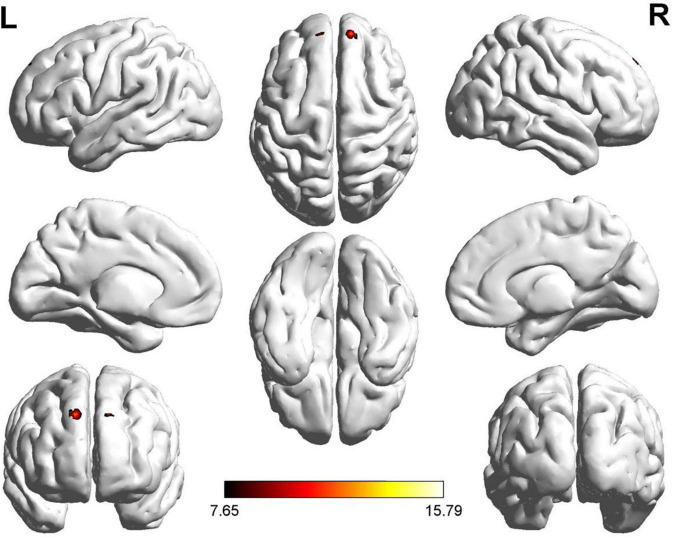
Significant differences in ALFF values were observed in the left and right dorsolateral SFG and rectus gyrus for both Recent-onset Tinnitus (ROT) and Persistent Tinnitus (PT) groups, compared to the healthy control group.

We found significant differences in ALFF values in the left and right dorsolateral superior frontal gyrus (SFG) and left gyrus rectus (GR) in the ROT and PT groups compared to those in the control group (Figure 1). Compared with the control group, the t-values of the ALFF for both the ROT and PT groups in the left GR were significantly lower than the global mean values from the control group (*p* < 0.05). The t-value of the ALFF in the ROT group was lower than that in the PT group (*p* < 0.05).

No statistically significant difference was observed for the left dorsolateral SFG between the ROT and PT groups (*p* > 0.05). However, compared with the control group, the ALFF t-values for both the ROT and PT groups in the right dorsolateral SFG were significantly lower than the global mean values from the control group (*p* < 0.05).

No statistically significant difference was observed for the right GR between the ROT and PT groups (*p* > 0.05). However, compared with the control group, the t-values of the ALFF for both the ROT and PT groups in the left GR were significantly lower than the global mean values from the control group (*p* < 0.05). The results are presented in Table 2A.

**TABLE 2A:** Decreased ALFF activities in both ROT and PT groups compared to the control group.

Cluster number	Cluster size (voxel)	Peak MNI coordinate			Peak MNI coordinate region	*F* value	T-value difference between ROT and PT	T-value difference between ROT and control group	T-value difference between PT and control group
		*X*	*Y*	*Z*					
1	25	–12	45	–15	Left gyrus rectus	14.36	–2.93	–5.18	–2.25
2	49	–15	54	42	Left dorsolateral SFG	15.79	No significant difference	–4.60	–4.34
3	73	15	54	45	Right dorsolateral SFG	13.85	No significant difference	–3.86	–4.26

MNI, Montreal Neurological Institute; ROT, recent-onset tinnitus; PT, persistent tinnitus; SFG, superior frontal gyrus; SPG, superior parietal gyrus.

### Regional homogeneity results

**FIGURE 2:**
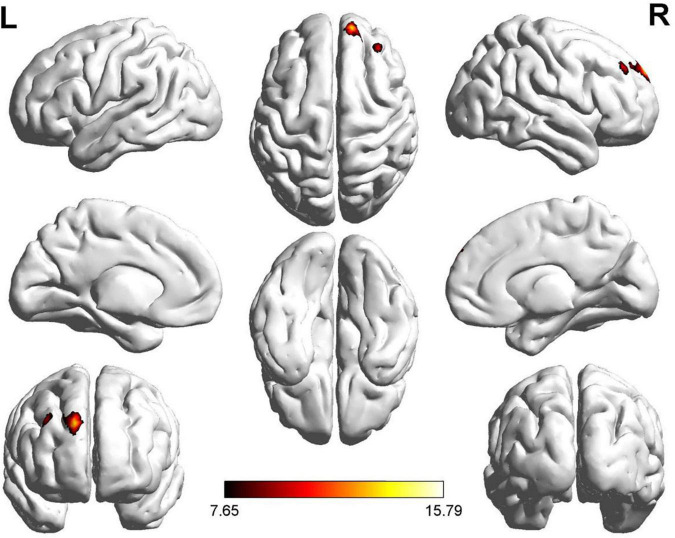
Significant differences in ReHo values were observed in the right dorsolateral SFG for recent-onset tinnitus (ROT) and persistent tinnitus (PT) groups, compared to the healthy control group.

We also discovered significant differences in ReHo values in the right dorsolateral SFG for both the ROT and PT groups compared with the control group (Figure 2). Regarding ReHo’s t-value, both the ROT and PT groups in the right dorsolateral SFG revealed significantly lower values than the global mean values in the control group (*p* < 0.05) (Table 2B). A two-sample t-test did not reveal any statistical differences between the ROT and PT groups in the right dorsolateral SFG (*p* > 0.05).

**TABLE 2B:** Decreased ReHo activities in both ROT and PT groups compared to the control group.

Cluster number	Cluster size (voxels)	Peak MNI coordinate			Peak MNI coordinate region	*F* value	T-value difference between ROT and PT	T-value difference between ROT and control group	T-value difference between PT and control group
		*X*	*Y*	*Z*					
1	160	39	45	39	Right dorsolateral SFG	10.02	No significant difference	–4.06	–4.77

MNI, Montreal Neurological Institute; ROT, recent-onset tinnitus; PT, persistent tinnitus; SFG, superior frontal gyrus; SPG, superior parietal gyrus.

### Voxel-wise functional connectivity results

**FIGURE 3:**
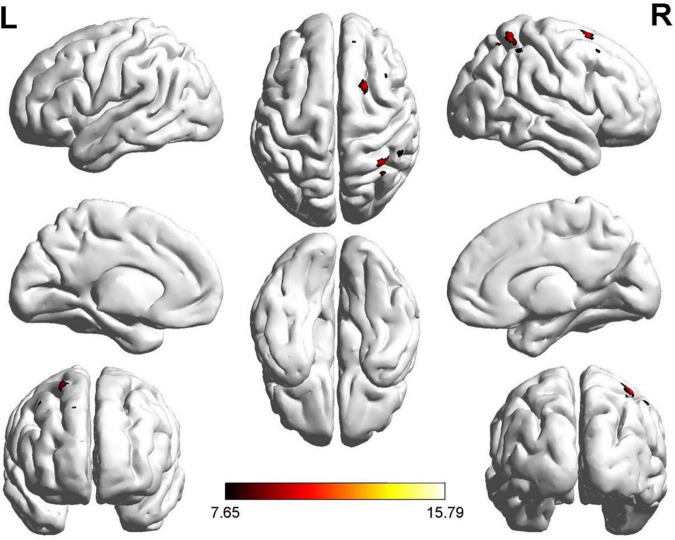
Significant FC patterns between the ROIs (dorsolateral SFG, left) and right SFG, left medial superior frontal gyrus (SFG), and right superior parietal gyrus (SPG) in the recent-onset tinnitus (ROT) and persistent tinnitus (PT) groups compared to the healthy control group.

**FIGURE 4:**
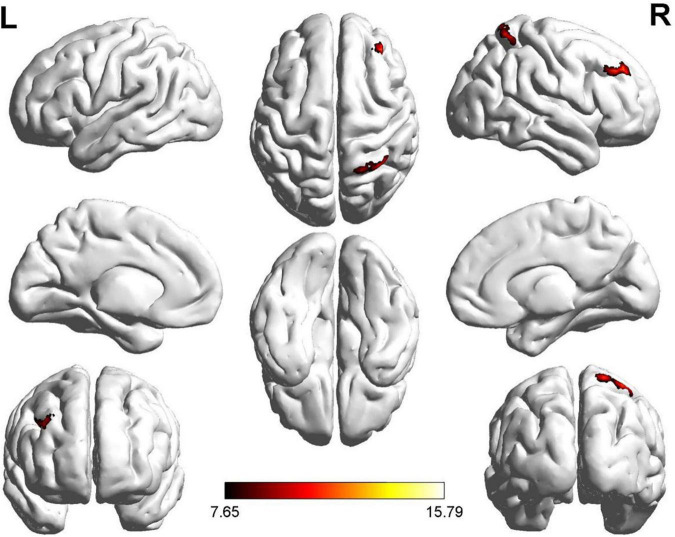
Significant FC patterns were observed between the right dorsolateral SFG and the right middle frontal gyrus (MFG), right medial superior frontal gyrus (SFG), and right superior parietal gyrus (SPG) in the recent-onset tinnitus (ROT) and persistent tinnitus (PT) groups compared to the CN healthy control group.

Two regions identified in the ALFF analysis (dorsolateral SFG, left and right) were used as seeds for further FC analysis. Brain regions with significant FC pattern differences for ALFF analysis in clusters 2 and 3 are shown in Figures 3, 4, respectively. In contrast to the control group, both the ROT and PT groups exhibited a reduction in connectivity between the seed region in the left dorsolateral SFG (ALFF cluster 2) and the right superior parietal gyrus (SPG), right dorsolateral SFG, and left medial SFG (*p* < 0.05) (Table 3A). No significant difference was observed between the ROT and PT groups (*P* > 0.05). At the same time, both the ROT and PT groups exhibited decreased connectivity between the seed regions in the right dorsolateral SFG (ALFF cluster 3) and right middle frontal gyrus (MFG), left medial SFG, and right SPG (*p* < 0.05) (Table 3B). No difference was observed between the ROT and PT groups (*p* > 0.05), except for a reduced connectivity pattern between the right dorsolateral SFG (ALFF cluster 3) and the right MFG in the PT group compared to the ROT group.

**TABLE 3A:** Decreased activities in voxel-wise FC ALFF cluster 2 for both ROT and PT groups than in the control group.

Cluster number	Cluster size (voxels)	Peak MNI coordinate			Peak MNI coordinate region	*F* value	T-value difference between ROT and PT	T-value difference between ROT and control group	T-value difference between PT and control group
		*X*	*Y*	*Z*					
1	150	45	–51	60	Right SPG	14.86	No significant difference	–3.78	–4.66
2	83	6	15	72	Right dorsolateral SFG	14.98	No significant difference	–3.62	–3.84
3	43	9	54	48	Left superior medial frontal gyrus	13.44	No significant difference	–3.36	–4.23

FC, functional connectivity; ROT, recent-onset tinnitus; PT, persistent tinnitus; SFG, superior frontal gyrus; SPG, superior parietal gyrus.

**TABLE 3B:** Decreased activities in voxel-wise FC ALFF cluster 3 for ROT and PT groups than in the control group.

Cluster number	Cluster size (voxels)	Peak MNI coordinate			Peak MNI coordinate region	*F* value	T-value difference between ROT and PT	T-value difference between ROT and control group	T-value difference between PT and control group
		*X*	*Y*	*Z*					
1	132	39	42	39	Right MFG	14.86	3.18	–4.58	–5.09
2	46	–3	42	57	Left medial superior frontal gyrus	14.98	No significant difference	–3.82	–4.60
3	93	36	–57	66	Right superior parietal gyrus	13.44	No significant difference	–4.36	–4.07

FC, functional connectivity; MFG, middel frontal gyrus; ROT, recent-onset tinnitus; PT, persistent tinnitus.

**FIGURE 5:**
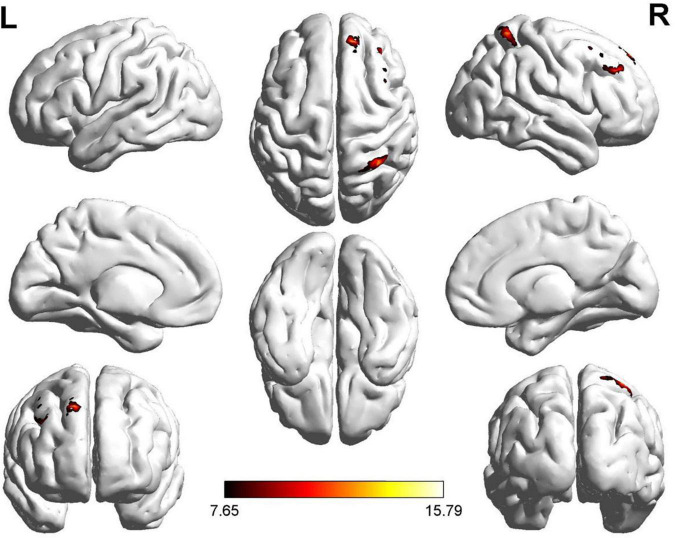
Significant FC patterns were observed between the right dorsolateral SFG and the right middle frontal gyrus (MFG), right superior parietal gyrus (SPG), and right dorsolateral SFG in the recent-onset tinnitus (ROT) and persistent tinnitus (PT) groups, compared to the healthy control group.

One region identified in the ReHo analysis (right dorsolateral SFG) was used as the seed for further FC analysis. The brain regions with significant FC pattern differences are shown in Figure 5. In contrast to the control group, both the ROT and PT groups demonstrated lower connectivity levels between the seed region in the right dorsolateral SFG and right MFG, left medial superior frontal gyrus, and right superior parietal gyrus (SPG) (*P* < 0.05) (Table 3C). No difference was observed between the ROT and PT groups (*p* > 0.05), except for an elevated connectivity pattern between the right dorsolateral SFG (ReHo Cluster 1) and right MFG in the ROT group compared to the PT group.

**TABLE 3C:** Decreased activities in voxel-wise functional connectivity (ReHo cluster 1) for both ROT and PT groups than in the control group.

Cluster number	Cluster size (voxels)	Peak MNI coordinate			Peak MNI coordinate region	*F* value	T-value difference between ROT and PT	T-value difference between ROT and control group	T-value difference between PT and control group
		*X*	*Y*	*Z*					
1	120	48	30	36	Right MFG	14.86	3.89	–2.79	–5.00
2	80	15	51	48	Right dorsolateral SFG)	14.98	No significant difference	–4.17	–4.66
3	96	36	–51	66	Right SPG	13.44	No significant difference	–3.31	–4.49

ROT, recent-onset tinnitus; PT, persistent tinnitus; SFG, superior frontal gyrus; SPG, superior parietal gyrus.

## Discussion

In the current study, we utilized various resting-state fMRI technologies, including ALFF, ReHo, and voxel-wise FC, to investigate the differences in intra-regional brain activity and inter-regional FC in patients with ROT and PT. To the best of our knowledge, this is the first study to reveal neuronal changes during the transition from ROT to PT using resting-state fMRI.

Our findings revealed that participants with ROT and PT demonstrated abnormal intra-regional neural activity and disrupted FC. In addition, regions of some non-auditory networks including the DMN, optical network, DAN, and CEN were affected (Chen et al., 2017). Furthermore, we discovered significant differences within the ALFF, ReHo, and FC activity levels between the ROT and PT groups, with the PT group demonstrating the lowest activity and connectivity levels among the three groups. In order to identify the differences in brain activity between recent-onset and PT participants, we explored the roles of each brain region revealed by the rs-FMRI analysis and identified possible strategies to prevent the transition from ROT to PT.

### Elevated activity in left gyrus rectus for persistent tinnitus patients

The GR is located on the medial margin of the inferior surface of the frontal lobe. Although its specific function remains unclear, clinical reports have indicated that patients who undergo surgical removal of the GR demonstrate temporary cognitive deficits, including a reduction in memory and personality changes (Joo et al., 2016). In addition, studies using resting-state FC technologies have revealed that patients with distressful tinnitus demonstrate abnormal brain activity within the bilateral GR (Ueyama et al., 2013, 2015). Furthermore, studies have also revealed that the GR demonstrates anatomical connections with the limbic system (Lan et al., 2022). Du et al. (2020) reported that the GR demonstrated strong FC with the anterior, medial, and posterior orbital gyrus, SFG, ventromedial prefrontal cortex, and anterior cingulate cortex (Du et al., 2020).

Our findings revealed that participants with ROT demonstrated reduced activity levels in the GR compared with participants with PT. In addition, compared with the healthy control group, participants from both tinnitus groups demonstrated reduced GR activity levels. Therefore, these results indicate that patients with recent-onset or PT might perceive temporary cognitive decline due to disruptions in the GR. Furthermore, for patients with ROT, the level of disruption to cognitive processing due to tinnitus may be higher than that in patients with PT due to the novelty of tinnitus.

### Reduced activity in dorsolateral superior frontal gyrus for both recent-onset and persistent tinnitus patient

Both ALFF and ReHo analyses revealed a reduction in activity level in the dorsolateral SFG on both sides in participants with PT or ROT. The results did not reveal significant differences in dorsolateral SFG activities between the recent-onset tinnitus (ROT) and PT groups. The main functions of the dorsolateral SFG are top-down processing and cognitive functions, including working memory, episodic memory, goal-driven attention, planning, problem solving, and task switching. These findings imply the role of the dorsolateral SFG in CEN manipulation (Kinoshita et al., 2012; Hu et al., 2016).

In addition, the dorsolateral SFG demonstrates FC with the DMN, especially with the precuneus. The existing literature indicates that the DMN specializes in internally oriented cognitive processes, such as conceptual processing, daydreaming, and future planning (Cloutman and Lambon Ralph, 2012; Lin et al., 2017). Therefore, we suggest that the dorsolateral SFG regulates the interaction between the CEN and DMN. Reduced dorsolateral SFG activity might disrupt the CEN, eventually reducing patients’ top-down attention-filtering capability. Furthermore, our results suggest that the overall duration of tinnitus does not contribute to reduced activity levels in the left and right dorsolateral SFG. Tinnitus patients can perceive difficulties switching their attention away from the tinnitus, regardless of experiencing recent-onset or PT.

### Reduced functional connectivity between bilateral dorsolateral superior frontal gyrus and medial superior frontal gyrus in both recent-onset and persistent tinnitus participants

The existing literature has revealed that the medial SFG has anatomical connections with the cingulate cortex (mainly the anterior and medial section of the cingulate cortex, ACC, and MCC) through the cingulum and that functional correlation with the MCC and the DMN (Nagahama et al., 1999). In addition, dense connections between the dorsolateral prefrontal cortex (DLPFC) (including the SFG) and, ACC, and MCC, have also been discovered in humans (Zhang et al., 2011; Cloutman and Lambon Ralph, 2012; Ueyama et al., 2013).

Moreover, rs-FCs between SFG, ACC, and MCC have been reported (Cloutman and Lambon Ralph, 2012; Yang et al., 2014; Khan et al., 2021). The anatomical and functional connections between the medial SFG and anterior MCC suggest that the medial SFG is involved in cognitive control because the anterior part of the MCC is related to cognitive control, including conflict monitoring, response selection, error detection, and attention manipulation. Additionally, the medial SFG demonstrates anatomic connections with the ACC, a core node of the DMN, and a functional correlation with the DMN, suggesting that the medial SFG is critical for DMN manipulation (Hu et al., 2021).

Our findings suggest that the overall duration of tinnitus onset does not play a role in generating FC differences in the left medial SFG. Nevertheless, participants from the ROT and PT groups demonstrated reduced FC between the bilateral dorsolateral SFG and left medial superior frontal gyrus compared with the healthy control group. Therefore, we propose that reduced FC between the dorsolateral SFG and medial SFG disrupts DMN regulation, further reducing the patients’ ability to manipulate attention. Furthermore, this significant change within the top-down attention-regulating mechanism increases tinnitus perception regardless of the overall duration of tinnitus onset.

### Functional connectivity abnormality between bilateral dorsolateral superior frontal gyrus and right middle frontal gyrus in both patients with recent-onset and persistent tinnitus

As a critical component of the VAN, the right MFG serves as a convergence center for the DAN and VAN by working as a circuit breaker to interrupt ongoing endogenous attentional processes in the DAN and reorient attention to exogenous stimuli (Japee et al., 2015; Briggs et al., 2021). Furthermore, the right MFG actively engages in reorienting distinctive signals from unexpected locations (Carter et al., 2006).

Our findings revealed reduced FC between the dorsolateral and right MFG. This change could lead to a disruption between the VAN and DAN, which influences attention orientation to novel stimuli. This conclusion agrees with the typical description of patients with tinnitus that they unconsciously perceive their tinnitus to be more prominent in quieter situations, regardless of the tinnitus duration (Xu et al., 2019).

In addition, we also discovered that participants with tinnitus developed within six months (ROT group) demonstrated statistically higher FC than participants with PT. Participants with PT also demonstrated higher THQ, SAS, and SDS scores than participants with ROT. This result indicated that patients with tinnitus would experience more difficulties reorienting their attention away from tinnitus once it lasted longer than six months (from recent-onset to persistent).

### Reduced functional connectivity between bilateral dorsolateral superior frontal gyrus and superior parietal gyrus in both recent-onset and persistent tinnitus participants

The main functions of the SFG are spontaneous attention regulation and top-down processing. The existing literature suggests that the SPG becomes more active during a task-free resting-state. Since the SFG acts as a critical component of the superior parietal lobule (SPL), it demonstrates a strong connection with the occipital lobe and involves somatosensory and visuospatial stimulus integration, written language, and working memory (Berlucchi and Vallar, 2018). The existing literature also reported SPG’s implications of SPG in shifting attention between visual targets and spatial-related attention shift states (Lin et al., 2021). Our findings revealed no significant differences between the FC levels in the ROT and PT groups. However, both groups demonstrated reduced FC compared with the healthy control group. Thus, this finding indicates that reduced FC between the dorsolateral SFG and SPG could disrupt working memory in patients with tinnitus, regardless of tinnitus duration.

### Clinical significance of our findings in tinnitus management

The existing literature indicates that the level of tinnitus distress within six months of the initial onset predicts the long-term level of tinnitus distress in patients after six months of onset. Patients who perceive higher levels of tinnitus disruption are more likely to develop persistent and debilitating tinnitus. Multiple findings from our study indicate that patients with ROT demonstrate reduced capability of top-down attention and stimuli monitoring and orientation. Therefore, clinicians should provide effective tinnitus management strategies for patients with ROT (Kleinstäuber and Weise, 2020).

Considering that the cause of tinnitus can be multifactorial, there is no standard treatment plan for tinnitus. Nevertheless, clinicians can effectively manage tinnitus using multidisciplinary options. According to clinical practice guidelines for tinnitus from the American Academy of Otolaryngology-Head and Neck Surgery, patient education and counseling, hearing amplification, sound therapy, and cognitive-behavioral therapy should be implemented individually or in combination for tinnitus management (Tunkel et al., 2014; Zenner et al., 2016; Liu et al., 2021; Osuji, 2021).

### Research limitations and room for improvement

The small sample size of our study may have reduced our ability to detect causal relationships between abnormal connectivity patterns and tinnitus characteristics. Therefore, studies with a larger sampling size are strongly recommended. Furthermore, subjects were exposed to equipment noises during the scanning process. Even though noise cancelation was instrumented in all subjects, scanner noise may reduce subjects’ tinnitus levels, thereby changing rs-FC status. Therefore, reducing the noise of brain imaging equipment will be helpful in future tinnitus-related investigations.

## Conclusion

Our project indicated a reduced activity level within the dorsolateral SFG (left and right) and GR using ALFF and ReHo analyses. Patients with PT demonstrated higher activity levels in the GR than those with ROT. Furthermore, our follow-up voxel-wise FC revealed decreased connection activity between the dorsolateral SFG (left and right) and right SPG, right MFG, and left mSFG for participants with ROT and PT, compared to the healthy control group. Patients with ROT demonstrated a higher level of FC than those with PT did. Our data suggest that patients with PT are more likely to experience difficulties in monitoring external stimuli and attention reorientation than patients with ROT. In addition, patients who perceive higher levels of tinnitus disruption are more likely to develop persistent and debilitating tinnitus. Therefore, we strongly recommend that clinicians implement effective tinnitus management strategies in patients with ROT as soon as possible.

## Data availability statement

The original contributions presented in this study are included in the article/supplementary material, further inquiries can be directed to the corresponding authors.

## Ethics statement

The studies involving human participants were reviewed and approved by the Research Ethics Committee of the Affiliated Zhongda Hospital of Southeast University. The patients/participants provided their written informed consent to participate in this study.

## Author contributions

HD: conceptualization, methodology, investigation, formal analysis, and writing—original draft preparation. XF: resources, investigation, data curation, and writing—review and editing. XQ: supervision, project administration, writing—review and editing, and funding acquisition. JZ: resources, software, validation, visualization, and formal analysis. BL: resources, software, validation, and formal analysis. AL: funding support, resources, and validation. XG and ZH: supervision, project administration, and funding acquisition. All authors contributed to the article and approved the submitted version.
